# Differential pathways from personality to risk-taking: how extraversion and negative emotionality shape decision-making through overconfidence

**DOI:** 10.3389/fpsyg.2025.1537658

**Published:** 2025-07-28

**Authors:** Yangxizhao He, Peng Lei

**Affiliations:** China Center for Behavioral Economics and Finance, Southwestern University of Finance and Economics, Chengdu, China

**Keywords:** personality traits, risk decision-making, overconfidence, mediation effect, Game of Dice Task, behavior economics

## Abstract

**Introduction:**

Understanding mechanisms through which personality traits influence risk decision-making remains crucial in behavioral research. This study examined overconfidence as a mediator in personality-risk relationships, focusing on Extraversion and Negative Emotionality.

**Methods:**

We recruited 110 university students to complete the Game of Dice Task, Big Five Inventory-2, and General Knowledge Questionnaire. Mediation analyses using bootstrapping methods examined direct and indirect effects with demographic controls.

**Results:**

Mediation analyses revealed distinct patterns. Extraversion demonstrated partial mediation through overconfidence (indirect effect: β = 0.101, 95% CI [0.012, 0.228]), explaining 20.5% of the total effect. Negative Emotionality showed complete mediation through overconfidence (indirect effect: β = –0.216, 95% CI [–0.364, –0.091]), accounting for 72.8% of the total effect. The models explained 29.4% and 22.9% of variance respectively.

**Discussion:**

These findings demonstrate that personality traits influence risk decisions through distinct pathways: Extraversion operates through both direct behavioral tendencies and cognitive biases, while Negative Emotionality primarily influences risk behavior through confidence calibration. Results provide implications for theoretical development and practical interventions, suggesting personality-tailored approaches to risk management.

## 1 Introduction

Risk decision-making, a fundamental aspect of human behavior, has profound implications across various life domains, from financial investment to career choices. The evolution of research in this field has progressed from identifying trait-behavior correlations to exploring underlying psychological mechanisms. This progression reflects a broader shift in personality psychology toward understanding not just what traits predict behavior, but how and why these predictions occur. Recent years have witnessed growing interest in understanding how personality traits shape these differences, particularly through cognitive mechanisms such as overconfidence. The relationship between personality and risk-taking has been well-documented, with traits like Extraversion and Negative Emotionality consistently emerging as significant predictors (Frey et al., 2017). Meanwhile, overconfidence has been identified as a crucial factor in risk-related decisions, often leading to systematic biases in judgment and choice (Moore and Schatz, 2017). Understanding how these psychological factors interact to influence risky decisions not only advances our theoretical knowledge but also provides practical insights for improving decision-making processes across various professional and personal contexts.

Research on personality and risk-taking has established robust foundations, particularly regarding traits like sensation-seeking and impulsivity. Zuckerman and Kuhlman (2000) demonstrated that risk-taking across multiple domains (smoking, drinking, drugs, sex, driving, gambling) is strongly related to impulsive sensation seeking, aggression, and sociability, revealing that personality traits underlying risk-taking behavior share common biological factors, including dopaminergic activity and specific genetic markers. Similarly, Nicholson et al. (2005) identified clear Big Five patterns for overall risk propensity, combining high extraversion and openness with low neuroticism, agreeableness, and conscientiousness, with sensation-seeking emerging as a key component of risk behavior.

Recent advances have further elucidated these relationships. Frey et al. (2017) provided compelling evidence for a general factor of risk preference (R) that emerges from stated preferences and generalizes to real-world risky activities, demonstrating high reliability across time and thus qualifying as a stable psychological trait. Capra et al. (2013) revealed that personality types (motivated, impulsive, and affective) systematically influence probability distortions in risky choice, with impulsive participants showing enhanced discrimination of non-extreme probabilities. Furthermore, Engelmann and Hirmas (2023) demonstrated that impulsiveness moderates attention effects in risky decision-making, with more impulsive individuals becoming more sensitive to attribute values when gains receive longer attention.

Despite extensive research on personality and risk decision-making, several critical gaps remain in our understanding. First, existing studies have primarily focused on direct relationships between personality traits and risk behavior (Nicholson et al., 2005; Lauriola and Levin, 2001), or between overconfidence and decision outcomes (Barber and Odean, 2001; Malmendier and Tate, 2005; Fast et al., 2012). While these direct relationships are important, they provide limited insight into the psychological mechanisms through which personality traits ultimately influence risky decisions. Recent theoretical developments suggest that trait-behavior relationships likely operate through multiple pathways (Baumert et al., 2017; Wood et al., 2015), yet empirical tests of these mediating processes remain scarce. This limitation has hindered our ability to develop comprehensive models of personality influence on decision-making.

Second, although some studies suggest that personality traits might influence risky decisions through cognitive pathways (Wilmot and Ones, 2019; Josef et al., 2016), the specific role of cognitive biases, particularly overconfidence, remains underexplored. This gap is notable given that overconfidence has been identified as a crucial factor in risk-related decisions across various domains (Moore and Healy, 2008; Cesarini et al., 2006). Understanding how personality traits interact with cognitive biases could provide valuable insights into the development and maintenance of risk-taking tendencies. The potential mediating role of overconfidence is particularly intriguing given its demonstrated influence on decision-making and its theoretical connections to personality traits. However, recent evidence questioning overconfidence as a stable individual difference (Li et al., 2025) raises important questions about its reliability as a mediating mechanism, necessitating careful examination of its role in personality-risk relationships.

Third, methodological limitations have constrained our understanding of these relationships. While Extraversion and Negative Emotionality have been consistently linked to risk behavior, the mechanisms underlying these relationships may differ substantially. Recent neuroimaging research suggests distinct neural pathways for different personality-risk relationships (Holmes et al., 2016), but behavioral evidence remains limited. Most studies have relied heavily on self-report measures rather than behavioral tasks, potentially introducing common method bias and limiting ecological validity. Additionally, few studies have employed robust experimental designs that can effectively distinguish between direct and indirect effects of personality on risk behavior (Frey et al., 2017; Figner and Weber, 2011). These methodological constraints have limited our ability to draw definitive conclusions about the causal pathways involved.

While extensive research has established personality-risk relationships, particularly for sensation-seeking and impulsivity traits, the specific cognitive mechanisms through which personality traits influence risky decisions remain underexplored. The differential pathways through which distinct personality dimensions (e.g., Extraversion vs. Negative Emotionality) may operate through cognitive biases require systematic investigation. The present study addresses these gaps by examining how personality traits influence risk decision-making through overconfidence, using both self-report measures and behavioral experiments. Building on the established foundation of personality-risk research, this investigation aims to elucidate the differential pathways through which personality traits affect risky decisions while employing methodologically robust approaches. By combining multiple measurement methods and focusing on specific mediating mechanisms, this research seeks to provide a more nuanced understanding of personality-risk relationships.

Understanding the psychological mechanisms underlying risk decision-making requires integrating perspectives from personality theory and cognitive psychology. The Five-Factor Model (FFM) provides a robust theoretical framework for understanding these variations (Costa and McCrae, 2008), with substantial evidence linking personality traits to risk-taking behaviors (Nicholson et al., 2005). Among the Big Five personality dimensions, Extraversion and Negative Emotionality represent theoretically and empirically distinct pathways to risk behavior. Schaefer et al. (2004) demonstrated that Extraversion significantly predicts overconfidence even when controlling for other Big Five factors, aligning with reinforcement sensitivity theory (RST) predictions about approach motivation and reward sensitivity. Meta-analytic evidence from Frey et al. (2017) confirms that these dimensions show the strongest and most consistent relationships with risk-taking across domains and measurement methods.

The selection of these specific traits is further justified by their theoretical distinctiveness: Extraversion primarily relates to approach motivation and sensation-seeking (Zuckerman and Kuhlman, 2000), while Negative Emotionality involves threat sensitivity and loss aversion (Nicholson et al., 2005). This conceptual separation allows for examining differential pathways through which personality traits may influence risky decisions via cognitive mechanisms. Particularly, Extraversion and Negative Emotionality have emerged as significant predictors through distinct mechanisms.

According to Gray’s (1970) RST, Extraversion relates to enhanced reward sensitivity and approach motivation. This theoretical perspective suggests that extraverted individuals are more attuned to potential rewards and less sensitive to punishment signals, leading to greater risk tolerance. Empirical evidence strongly supports this relationship, with studies demonstrating positive correlations between Extraversion and risky choices (Buelow and Cayton, 2020), and meta-analyses confirming this association (*r* = 0.31, *p* < 0.001; Wang et al., 2022). Furthermore, extraverted individuals typically exhibit stronger sensation-seeking tendencies and elevated dopaminergic activity (Depue and Collins, 1999), making them more susceptible to potential high rewards.

Conversely, Negative Emotionality relates to enhanced loss sensitivity and risk aversion (Hirsh and Inzlicht, 2008). This relationship operates through heightened attention to potential negative outcomes and increased sensitivity to uncertainty. Neuroimaging evidence suggests distinct neural pathways for these relationships (Botvinik-Nezer et al., 2020), with Negative Emotionality being associated with increased activation in threat-processing neural circuits during decision-making tasks.

Overconfidence has emerged as a crucial cognitive mechanism in risk-related decisions across diverse domains. Classical economic research has demonstrated its profound impact on financial behavior: Barber and Odean (2001) found that male investors’ overconfidence leads to 45% more trading than women, reducing returns by 2.65 percentage points annually. Similarly, Malmendier and Tate (2005) showed that overconfident CEOs overinvest when having abundant internal funds but curtail investment when requiring external financing, significantly affecting corporate investment decisions.

The phenomenon extends beyond simple confidence miscalibration to fundamental metacognitive deficits. The Dunning-Kruger effect (Kruger and Dunning, 1999) reveals that individuals with limited competence not only reach erroneous conclusions but also lack the metacognitive ability to recognize their incompetence, leading to systematic overestimation of performance. Stone (1994) further demonstrated that overconfident initial self-efficacy judgments affect decision processes and performance in complex cognitive tasks, with overconfidence paradoxically reducing effort and attention to strategy. Defined as excessive estimation of one’s abilities or judgments, overconfidence influences risky decisions through cognitive bias (Kahneman and Tversky, 1979) and illusion of control (Langer, 1975). Laboratory studies have demonstrated that experimentally induced overconfidence leads to increased risk-taking (Fast et al., 2012), while meta-analytic evidence confirms its positive relationship with risk-taking behaviors (Moore and Schatz, 2017). This effect appears particularly pronounced in tasks with explicit probability structures like the Game of Dice Task (GDT; Brand et al., 2007).

The relationship between personality traits and overconfidence provides a potential mediating mechanism. Trait Activation Theory (Tett and Burnett, 2003) suggests that personality traits systematically influence information processing, including confidence judgments. Research has demonstrated positive associations between Extraversion and overconfidence (Anderson et al., 2012), potentially due to extraverts’ tendency toward positive self-evaluation and their greater exposure to social reinforcement. Negative Emotionality shows more complex patterns (Tamir and Robinson, 2016), with some evidence suggesting context-dependent effects on confidence judgments.

Based on this theoretical framework and empirical evidence, we propose:

H1a: Extraversion positively influences risk preference in the GDT.

H1b: Negative Emotionality negatively influences risk preference in the GDT.

H2: Overconfidence positively influences risk preference in the GDT.

H3a: Extraversion exerts an indirect effect on GDT risk preference through overconfidence.

H3b: Negative Emotionality exerts an indirect effect on GDT risk preference through overconfidence.

This research aims to construct and validate a mediational model of “personality traits-overconfidence-risk decision-making,” investigating the underlying mechanisms through which personality traits (Extraversion and Negative Emotionality) influence individual risk decision-making via overconfidence. At the theoretical level, this research integrates personality psychology and behavioral decision theory, elucidating mechanisms of individual differences in risk decision-making and enriching the theoretical framework of personality influences on decision behavior (Johnson et al., 2020). At the practical level, this study provides empirical foundations for developing individualized guidance in domains such as financial investment and career selection, while offering references for relevant intervention program development (Weber and Johnson, 2022).

## 2 Methodology

### 2.1 Participants and procedure

Based on previous research examining personality-risk relationships (Wang et al., 2022) and mediation effects in decision-making studies (Hayes, 2018), we anticipated medium effect sizes (f^2^ = 0.15 for multiple regression analyses; *d* = 0.5 for mean comparisons). *A priori* power analysis using G*Power 3.1 (Faul et al., 2009) indicated that a minimum sample size of 92 participants would be required to detect medium-sized effects (f^2^ = 0.15) in our primary analyses (multiple regression with 5 predictors, α = 0.05, power = 0.80). For mediation analyses, Monte Carlo simulations following Fritz and MacKinnon’s (2007) recommendations suggested a minimum sample size of 78 participants to detect mediated effects with medium-sized α and β paths (power = 0.80). To account for potential data loss and ensure adequate statistical power, we aimed to recruit 120 participants.

This study was conducted at a university in Chengdu, Sichuan Province, utilizing E-Prime software-based tasks and paper-pencil tests. Initially, 120 participants were recruited randomly by poster. After excluding 10 participants due to missing questionnaire data and experimental procedure errors, data from 110 valid participants were included in the final analysis. Among the participants, 34.55% were from rural areas, and 65.45% were female. The mean age was 19.85 years (SD = 1.12). Participants were distributed across all 4 years of undergraduate study, with first-year students comprising the majority. Their major fields of study primarily included education, psychology, and biology, with none having backgrounds in economics or management.

The research protocol strictly adhered to the ethical guidelines for human subjects research outlined in the Declaration of Helsinki and was approved by the Research Ethics Committee of the Sichuan Psychological Society. Prior to participation, all participants provided written informed consent. They were informed that their experimental compensation would be performance-based to ensure adequate motivation. During the experiment, participants retained the right to withdraw from the study, request the removal of their data, and control access to subsequent analytical results. The final compensation was determined by the remaining tokens in the GDT, consisting of a fixed participation fee plus performance-based incentives. The average total compensation was 45.4 yuan (SD = 12.5).

### 2.2 Research tools

#### 2.2.1 Game of dice task

This research employs the GDT, developed by Brand et al. (2005), to measure individual risk decision-making tendencies. The GDT represents a valuable alternative to the more commonly used Iowa Gambling Task (IGT) for investigating risk-related decision-making. While the IGT employs ambiguous feedback and implicit learning of reward-punishment contingencies, the GDT provides explicit probability structures that allow participants to make informed decisions based on clear risk-reward relationships (Schiebener and Brand, 2015). This explicit structure is particularly advantageous for examining cognitive influences on risky decisions, as it enables participants to engage in deliberate risk evaluation rather than relying primarily on affective feedback learning.

The GDT is a computerized decision task with explicit rules and probability structures, widely applied in risk decision-making research (Brand et al., 2008; Schiebener and Brand, 2015). Participants begin with 1,000 units of virtual currency and complete 18 rounds of dice roll predictions. Before each round, participants choose from four options with varying risk levels: single number prediction (1/6 winning probability, 1:1000 odds), two-number combination (2/6 probability, 1:500 odds), three-number combination (3/6 probability, 1:200 odds), or four-number combination (4/6 probability, 1:100 odds). Immediate feedback follows each decision, including dice roll outcome, profit/loss amount, and current total.

Following Brand et al.’s (2009) risk classification criteria, selections of single numbers or two-number combinations (winning probability ≤ 33%) constitute high- risky decisions, while three- or four-number combinations (winning probability ≥ 50%) represent low- risky decisions. This classification system has received extensive validation in subsequent research. Primary outcome measures include the proportion of high-risk choices (high-risk selections/total decisions), final virtual currency amount, and decision strategy stability (maximum consecutive uses of the same option). The explicit probability structure of the GDT makes it particularly suitable for investigating cognitive mechanisms such as overconfidence, as participants can form clear expectations about outcomes and confidence in their decisions.

Implementation follows a standardized protocol: detailed rule explanation with comprehension verification, two practice rounds, and 18 formal decision trials. Given our research objectives, we employ the proportion of high-risk choices as the primary dependent variable, an indicator demonstrated to effectively reflect individual risk preferences. Previous research confirms this measure’s strong psychometric properties and significant correlations with actual risk behaviors (Brand et al., 2009). Meta-analytic findings (Schiebener and Brand, 2015) suggest that detecting correlations between individual difference variables and GDT performance requires minimum sample sizes of 64–82 participants (α = 0.05, power = 0.80), making this task well-suited for individual difference research.

#### 2.2.2 Big five inventory-2

The Five-Factor Model represents one of the most widely utilized and empirically supported theoretical frameworks in personality research (McCrae and Costa, 2008). This study employs the Big Five Inventory-2 (BFI-2) developed by Soto and John (2017), a refined version of the classic Big Five Inventory optimized through large-scale cross-cultural research data. The BFI-2 addresses several limitations of earlier personality measures, demonstrating enhanced structural and predictive validity across diverse populations.

The BFI-2 comprises 60 items rated on a 5-point Likert scale (1 = “strongly disagree” to 5 = “strongly agree”). The instrument assesses five personality dimensions: Extraversion, Agreeableness, Conscientiousness, Negative Emotionality, and Open-Mindedness, with 12 items per dimension. The present investigation focuses specifically on the Extraversion and Negative Emotionality dimensions, selected based on their theoretical relevance to risk decision-making processes.

The BFI-2 demonstrates excellent psychometric properties across multiple validation studies. Initial development studies reported internal consistency coefficients (Cronbach’s α) ranging from 0.88 to 0.90 and test-retest reliability coefficients ranging from 0.76 to 0.84 (Soto and John, 2017). Cross-cultural research has confirmed the instrument’s robust psychometric properties across various language versions (Denissen et al., 2020). The Chinese version of the BFI-2, developed through rigorous translation-back-translation procedures and validated across multiple Chinese samples (*N* > 1,000), demonstrates strong structural and criterion-related validity, with internal consistency coefficients ranging from 0.81 to 0.86 (Liu et al., 2021). In the current study, Cronbach’s α coefficients for Extraversion and Negative Emotionality are 0.85 and 0.83, respectively, consistent with previous validation studies.

#### 2.2.3 General knowledge questionnaire

This research employs the interval estimation method using the General Knowledge Questionnaire (GKQ) to measure overconfidence. The interval estimation approach, initially proposed by Alpert and Raiffa (1982) and subsequently validated in overconfidence research (Moore and Healy, 2008; Soll and Klayman, 2004), provides a robust method for assessing overprecision—the tendency to be overly certain about the accuracy of one’s knowledge. This method requires participants to provide interval estimates for factual questions with instructions to be 90% confident that the true value falls within their specified range. Compared to point estimation methods, interval estimation offers advantages of simpler administration, more objective scoring, and reduced susceptibility to response bias (Yaniv and Foster, 1997).

Considering cultural differences and contemporary relevance for Chinese participants, we developed a culturally adapted version. The adapted questionnaire includes items such as:

• World population as of January 2018 (approximately 7.59 billion)
• Mozart’s birth year (1756)
• Number of OPEC member countries (13)

(The complete questionnaire is provided in Appendix 1.)

The standardized scoring procedure calculates overconfidence by counting the number of items for which the true value falls outside participants’ confidence intervals. Under well-calibrated judgment with 90% confidence instructions, approximately 10% of items (about 1 out of 10) should fall outside the estimated intervals. However, research consistently demonstrates that most participants provide overly narrow intervals, resulting in significantly higher scores than this theoretical ideal, indicating systematic overconfidence (Klayman et al., 1999). In our pilot sample (*N* = 50), the adapted GKQ demonstrated test-retest reliability of 0.82, comparable to the original version, with mean difficulty indices equivalent to the original scale (adapted = 0.48, original = 0.45).

### 2.3 Statistical analysis

Following data collection, we conducted comprehensive data cleaning and processing for each experimental component. This included computing Big Five personality scores, calculating overconfidence measures, and deriving risk preference indices from the GDT to create the final dataset. Statistical analyses were performed using SPSS version 25.0 (IBM Corp, 2017). Our analytical strategy comprised three main components: descriptive statistics, difference tests, and mediation analyses, aimed at examining the relationships among personality traits, overconfidence, and risk preferences.

For the mediation analyses, we followed the procedures recommended by Hayes (2018) using the PROCESS macro (Model 4), which employs bootstrapping methods to test indirect effects. This approach is particularly robust for examining the mediating role of overconfidence in the relationship between personality traits and risk preferences. We used 5,000 bootstrap samples to generate 95% confidence intervals for the indirect effects, as recommended by current methodological guidelines (MacKinnon et al., 2012).

## 3 Result

### 3.1 Descriptive statistics

Preliminary analyses revealed key patterns among study variables (detailed distributions shown in Appendix 2). Participants demonstrated an overall risk-averse tendency, with negative mean values for both risk-seeking behavior (*M* = −6.95, SD = 10.38) and final GDT scores (*M* = −359.09, SD = 2496.17). Overconfidence levels were moderately high (*M* = 6.04, SD = 1.26) relative to the theoretical range (0–10). Among personality traits, Agreeableness showed the highest mean score (*M* = 45.28, SD = 6.11), while Extraversion (*M* = 38.12, SD = 7.06), Negative Emotionality (*M* = 39.25, SD = 7.04), and Conscientiousness (*M* = 39.20, SD = 6.29) demonstrated comparable levels the whole descriptive result has been shown in Table 1.

**TABLE 1:** Descriptive Statistics and intercorrelations among study variables.

Variable	*M*	SD	1	2	3	4	5	6	7
Risk seeking	−6.95	10.38	–						
Overconfidence	6.04	1.26	0.41**	–					
Extraversion	38.12	7.06	0.32**	0.21*	–				
Negative emotionality	39.25	7.04	−0.16	−0.38**	0.02	–			
Openness	40.69	6.92	−0.07	0.11	0.13	0.05	–		
Agreeableness	45.28	6.11	−0.1	−0.1	0.01	−0.07	−0.14	–	
Conscientiousness	39.2	6.29	−0.05	−0.07	0.05	−0.17	−0.17	0.51**	–

M and SD represent mean and standard deviation, respectively. The potential range for Risk Seeking: −18 to + 18; Overconfidence: 0–10; all personality measures: 12–60. *p < 0.05. **p < 0.01.

Distribution analyses revealed that risk preference scores exhibited a left-skewed pattern (skewness = −0.723, SE = 0.230), confirming the general risk-averse tendency. Overconfidence scores approximated normality (skewness = 0.156, SE = 0.230; kurtosis = −0.327, SE = 0.457), consistent with previous research on confidence calibration measures (Moore and Healy, 2008).

Correlation analyses revealed significant relationships supporting our theoretical framework. Risk-seeking behavior correlated positively with overconfidence (*r* = 0.41, *p* < 0.01) and Extraversion (*r* = 0.32, *p* < 0.01). Overconfidence demonstrated a positive association with Extraversion (*r* = 0.21, *p* < 0.05) and a negative association with Negative Emotionality (*r* = −0.38, *p* < 0.01). Among personality traits, Agreeableness and Conscientiousness showed a strong positive correlation (*r* = 0.51, *p* < 0.01), while Openness exhibited no significant associations with other variables. These correlational patterns provide initial evidence for the hypothesized personality-overconfidence-risk relationships.

### 3.2 Difference analysis

To examine demographic differences in risk-seeking behavior, we conducted a 2 × 2 factorial ANOVA with gender (male vs. female) and hukou status (urban vs. rural) as between-subjects factors. This approach provides a more comprehensive analysis of demographic effects while reducing multiple testing concerns compared to separate *t*-tests. The analysis revealed significant main effects for both demographic variables, with no significant interaction effect (see Table 2).

**TABLE 2:** Two-way ANOVA results for risk-seeking behavior.

Source	SS	df	MS	*F*	*p*	η^2^
Gender	1997.07	1	1997.07	24.37	<0.001	0.187
Hukou	761.55	1	761.55	9.29	0.003	0.081
Gender × Hukou	10.05	1	10.05	0.12	0.727	0.001
Error	8685.49	106	81.94			
Total	17048.00	110				

SS = sum of squares; MS = mean square. R^2^ = 0.260 (Adjusted R^2^ = 0.239).

**FIGURE 1:**
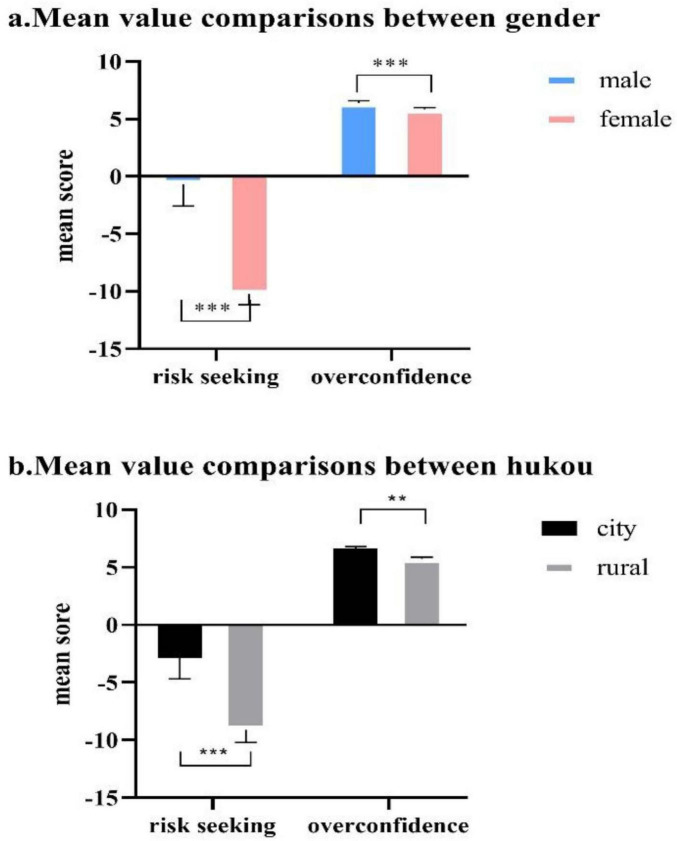
Mean value comparison for risk seeking and overconfidence between gender and hukou. *p < 0.05; **p < 0.01; ***p < 0.001. **(a)** The pink label means female gender, blue label means male gender. **(b)** The black label means City Hukou, gray label means Rural Hukou.

The main effect of gender was significant, F(1, 106) = 24.37, *p* < 0.001, η^2^ = 0.187, indicating substantial differences between male and female participants. Male participants (*M* = −0.68, SD = 11.61) demonstrated significantly higher risk-seeking tendencies compared to female participants (*M* = −10.25, SD = 7.94), with a mean difference of 9.57 points as shown in Figure 1a. This represents a large effect size according to Cohen’s conventions, suggesting that gender accounts for approximately 18.7% of the variance in risk-seeking behavior.

The main effect of hukou status was also significant, F(1, 106) = 9.29, *p* = 0.003, η^2^ = 0.081, representing a medium effect size. Urban residents (*M* = −2.84, SD = 11.29) exhibited significantly higher risk-seeking tendencies compared to rural residents (*M* = −9.11, SD = 9.23), with a mean difference of 6.27 points as shown in Figure 1b. This demographic factor explained approximately 8.1% of the variance in risk-seeking behavior, indicating a meaningful but moderate contribution.

The interaction between gender and hukou status was not significant, F(1, 106) = 0.12, *p* = 0.727, η^2^ = 0.001, indicating that the effects of gender and hukou status on risk-seeking behavior operate independently. This suggests that the gender difference in risk-seeking remains consistent across both urban and rural populations, and conversely, the urban-rural difference is similar for both male and female participants.

**FIGURE 2:**
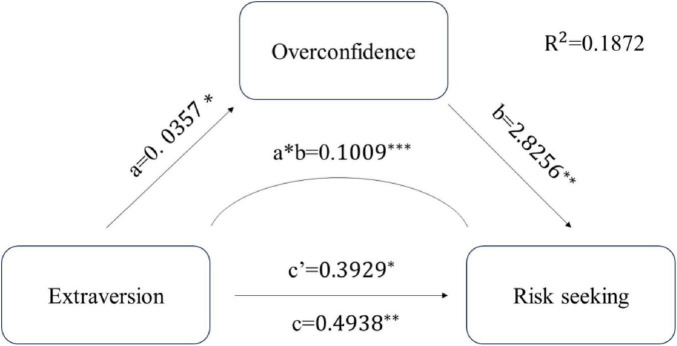
Path coefficient of extraversion model. *p < 0.05; **p < 0.01; ***p < 0.001.

The overall model explained 26.0% of the variance in risk-seeking behavior, F(3, 106) = 12.43, *p* < 0.001, indicating that a substantial proportion of individual differences can be attributed to these demographic characteristics. The demographic differences are visually represented in Figure 2, which displays group means with standard error bars for both risk-seeking behavior and overconfidence measures across all demographic categories.

For overconfidence measures, we conducted parallel analyses using the same factorial design. Gender showed a significant main effect, F(1, 106) = 4.89, *p* = 0.029, η^2^ = 0.044, with male participants (*M* = 6.39, SD = 1.22) demonstrating higher overconfidence levels than female participants (*M* = 5.85, SD = 1.24). Hukou status also demonstrated a significant main effect, F(1, 106) = 13.25, *p* < 0.001, η^2^ = 0.111, with urban residents (*M* = 6.61, SD = 1.20) showing higher overconfidence than rural residents (*M* = 5.74, SD = 1.19). The interaction effect was not significant, F(1, 106) = 0.28, *p* = 0.598, η^2^ = 0.003.

These findings reveal substantial demographic differences in both risk-seeking behavior and overconfidence. Notably, while both gender and hukou status showed significant effects across both measures, gender emerged as the stronger predictor for risk-seeking behavior, whereas hukou status showed comparable or stronger effects for overconfidence. The negative mean values across all groups for risk-seeking behavior indicate an overall risk-averse tendency in the sample, though this tendency was more pronounced among female and rural participants. The independence of gender and hukou effects suggests that demographic factors contribute additively to individual differences in decision-making behavior, providing important context for understanding personality-risk relationships in subsequent analyses.

### 3.3 Mediation analysis

To test the hypothesized mediating role of overconfidence in the relationship between personality traits and risk decision-making, we conducted parallel mediation analyses using the PROCESS macro (Model 4; Hayes, 2018) with 5,000 bootstrap samples. All analyses controlled for demographic variables (gender, age, hukou status, and monthly expenditure). Detailed results are presented in Appendix 3.

For the Extraversion model, results revealed a significant total effect of Extraversion on risk decision-making (β = 0.494, SE = 0.131, *p* < 0.001, 95% CI [0.234, 0.754]). After accounting for overconfidence as a mediator, both the direct effect (β = 0.393, SE = 0.126, *p* = 0.002, 95% CI [0.144, 0.642]) and indirect effect (β = 0.101, SE = 0.056, 95% CI [0.012, 0.228]) remained significant, indicating partial mediation. The mediation pathway operated through Extraversion positively predicting overconfidence (β = 0.036, SE = 0.017, *p* = 0.036), which subsequently predicted increased risk-seeking behavior (β = 2.826, SE = 0.717, *p* < 0.001). Overconfidence accounted for 20.5% of the total effect of Extraversion on risk decision-making, the path Figure as Figure 2.

**FIGURE 3:**
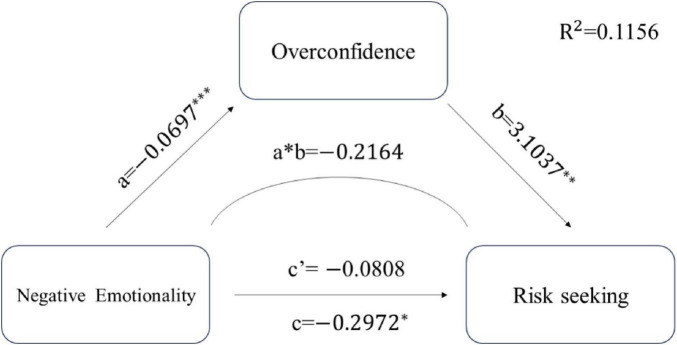
Path coefficient of negative emotionality model. *p < 0.05; **p < 0.01; ***p < 0.001.

For the Negative Emotionality model, analyses revealed a significant total effect on risk decision-making (β = −0.297, SE = 0.139, *p* = 0.034, 95% CI [−0.572, −0.023]). Critically, the direct effect became non-significant after including overconfidence as a mediator (β = −0.081, SE = 0.141, *p* = 0.569, 95% CI [−0.361, 0.200]), while the indirect effect remained significant (β = −0.216, SE = 0.070, 95% CI [−0.364, −0.091]), indicating complete mediation. The mediation pathway operated through Negative Emotionality negatively predicting overconfidence (β = −0.070, SE = 0.016, *p* < 0.001), which then predicted increased risk-seeking behavior (β = 3.104, SE = 0.798, *p* < 0.001). Overconfidence mediated 72.8% of the total effect of Negative Emotionality on risk decision-making, the path figure as Figure 3.

Effect size analyses using the kappa-squared (κ^2^) metric demonstrated moderate mediation strength for Extraversion (κ^2^ = 0.068) and large mediation strength for Negative Emotionality (κ^2^ = 0.143). The Extraversion model explained 29.4% of variance in risk decision-making (R^2^ = 0.294, F(6, 103) = 7.140, *p* < 0.001), while the Negative Emotionality model accounted for 22.9% of variance (R^2^ = 0.229, F(6, 103) = 5.099, *p* < 0.001). Both models demonstrated adequate statistical power (>0.80) and remained robust when controlling for demographic variables and potential outliers. These findings provide strong evidence for overconfidence as a key cognitive mechanism through which personality traits influence risk decision-making, with particularly pronounced mediation effects for Negative Emotionality.

## 4 Discussion

### 4.1 Main results

This investigation examined the intricate relationships among personality traits, overconfidence, and risk decision-making behavior. Our analyses yielded results that largely supported the proposed hypotheses while revealing nuanced patterns that enhance our understanding of personality-risk relationships.

Analysis of direct effects between personality traits and risk preference provided strong support for our first hypothesis (H1a), demonstrating a significant positive relationship between Extraversion and risk preference (β = 0.321, *p* < 0.01). The moderate to strong effect size (Cohen’s d = 0.628) aligns with predictions derived from RST (Gray and McNaughton, 2000), indicating that highly extraverted individuals consistently exhibit greater risk-taking propensity across decision contexts.

Our second hypothesis (H1b) regarding Negative Emotionality’s influence on risk preference received partial support. Although the direct effect trended in the expected negative direction (β = −0.158, *p* = 0.099), it did not reach conventional significance levels. However, subsequent analyses revealed more sophisticated relationships operating through indirect pathways, suggesting that Negative Emotionality’s influence on risky decisions involves more complex mechanisms than initially theorized. This finding extends existing literature by emphasizing the importance of examining indirect mechanisms in personality-risk relationships.

The hypothesized positive influence of overconfidence on risk preference (H2) received robust support (β = 0.411, *p* < 0.001). The consistency of this association across demographic groups and decision contexts suggests that overconfidence represents a stable psychological mechanism in risk decision-making, contributing to the growing evidence for the role of cognitive biases in financial decision-making (Moore and Healy, 2008).

Perhaps our most compelling finding concerns the mediating role of overconfidence (H3), which revealed distinct patterns across personality traits. Extraversion demonstrated partial mediation (indirect effect: β = 0.101, 95% CI [0.012, 0.228]), indicating that while overconfidence partially explains the relationship, direct effects remain significant. In contrast, Negative Emotionality exhibited full mediation through overconfidence (indirect effect: β = −0.216, 95% CI [−0.364, −0.091]), suggesting its influence operates primarily through confidence levels.

### 4.2 Theoretical implications

This investigation contributes to our understanding of personality-risk relationships and their underlying mechanisms in several ways. Our findings both support and extend existing theoretical frameworks while providing insights into how personality traits influence decision-making behavior.

First, we identified distinct pathways through which different personality traits influence risky decisions. Specifically, Extraversion demonstrated a positive relationship with risk-taking behavior (β = 0.321, *p* < 0.01), aligning with research on approach motivation and reward sensitivity (Smillie et al., 2011). This relationship operates through both direct and indirect pathways, supporting recent developments in personality neuroscience regarding multiple neural systems in trait expression (DeYoung, 2015). The partial mediation through overconfidence extends findings from organizational contexts (Zhang et al., 2021) to broader decision domains, suggesting that approach-oriented traits influence behavior through both automatic and controlled processes (Corr and Matthews, 2020). These findings align with the broader literature on sensation-seeking and impulsivity (Zuckerman and Kuhlman, 2000), demonstrating that our focus on Extraversion captures fundamental approach-motivated risk tendencies that have been consistently documented across multiple personality frameworks. Additionally, neuroimaging evidence indicates that extraverted individuals show enhanced activation in reward-processing regions during risk-taking tasks (Tompson et al., 2018), providing a potential neural basis for these behavioral patterns.

Second, our investigation revealed complexity in the relationship between Negative Emotionality and risky decisions. While previous research emphasized direct emotional influences on risk perception (Loewenstein et al., 2001), our findings indicate a mechanism operating primarily through cognitive appraisal processes. The full mediation through overconfidence (indirect effect: β = −0.216, 95% CI [−0.364, −0.091]) suggests that emotional dispositions influence decision-making predominantly through cognitive pathways. This finding contributes to the ongoing discussion about emotion-cognition relationships in decision processes. Recent neurobiological research has identified distinct neural circuits associated with negative emotionality and risk processing investigating the neural correlates of emotion regulation during decision-making tasks (Engelmann et al., 2018; Bertolacci et al., 2022), suggesting potential biological mechanisms underlying these relationships. Our results support an integrated model where emotional traits operate through cognitive processes, consistent with developments in emotional intelligence research (Joseph and Newman, 2010) and cognitive appraisal theory (Scherer and Moors, 2019).

Third, our findings illuminate specific cognitive mechanisms through which personality traits influence risky decisions, particularly the role of attention bias as a mediating pathway. Building on Engelmann and Hirmas (2023), who demonstrated that impulsiveness moderates attention effects in risky decision-making, our results suggest that overconfidence may operate through selective attention processes. Specifically, overconfident individuals may exhibit enhanced attention to information that supports their beliefs, such as focusing on potential gains rather than associated losses, or emphasizing potential outcomes rather than the risks involved. This attention bias mechanism provides a concrete cognitive pathway linking personality traits to risky decisions: extraverted individuals may develop overconfidence partly through their tendency to attend to reward-relevant information, while those high in Negative Emotionality may exhibit reduced overconfidence due to heightened attention to threat-relevant cues. This attention-based mechanism extends the Dunning-Kruger effect (Kruger and Dunning, 1999) by suggesting that personality traits systematically influence not only confidence calibration but also the information processing patterns that underlie confidence judgments. Supporting this attention-based framework, empirical evidence from eye-tracking studies demonstrates that individual differences in attentional allocation during risky decisions can significantly influence choice behavior, with attention to gains versus losses serving as a key moderating factor in risky decision-making (Hoven et al., 2023).

Fourth, the identification of overconfidence as a mediating mechanism across personality dimensions extends our theoretical understanding. This pattern suggests that overconfidence serves as a pathway through which personality traits influence risky decisions, building on previous work on overconfidence in financial decision-making. Our findings illuminate specific personality antecedents and their differential effects, contributing to understanding how cognitive biases influence decision-making processes. This extends seminal economic research by Barber and Odean (2001) and Malmendier and Tate (2005), who demonstrated overconfidence effects in investment behavior, by identifying the personality foundations of such cognitive biases. Recent meta-analytic evidence supports the role of overconfidence in risk-related decisions (Zhang et al., 2021), and our results extend this by identifying personality traits as important antecedents.

Fifth, our findings highlight the importance of decision-making competence as an individual difference variable. Consistent with research by Weller et al. (2018) on individual differences in decision-making styles, our results suggest that personality traits contribute to systematic variations in decision quality through their influence on confidence calibration. This perspective emphasizes that effective decision-making involves not only appropriate risk assessment but also accurate metacognitive awareness of one’s capabilities and limitations (Ceschi et al., 2025).

These findings develop personality and decision-making theory in several ways. They demonstrate that personality traits operate through multiple pathways (Roberts et al., 2007), emphasize the role of cognitive mechanisms in personality-behavior relationships (Hampson, 2012), and identify trait-specific pathways in decision-making processes (Corr, 2013). The results align with meta-analytic evidence on personality-behavior relations (Wilmot and Ones, 2019) while extending research on decision-making mechanisms. Additionally, our findings contribute to the broader theoretical framework of personality psychology by illustrating how traits manifest through both direct behavioral tendencies and cognitive processes.

### 4.3 Practical implications

Our findings have significant implications for various applied settings. The differential pathways through which personality traits influence risky decisions suggest the need for tailored approaches to risk management and decision support. For instance, interventions targeting extraversion-related risk-taking might need to address both direct behavioral tendencies and overconfidence, while interventions for individuals high in negative emotionality might focus primarily on calibrating confidence levels.

These insights could substantially inform professional practice across several domains, particularly in financial services where overconfidence has been shown to significantly impact investment outcomes. Building on the economic research tradition established by Barber and Odean (2001), practitioners could develop personality-informed risk assessment tools that account for both trait-level risk preferences and confidence calibration tendencies. Understanding clients’ personality profiles could help advisors better calibrate risk assessment and portfolio recommendations, leading to more personalized investment strategies. Similarly, in organizational settings, leadership development programs could incorporate personality-aware approaches to decision-making training, potentially improving team composition and risk management protocols. The findings also suggest implications for clinical applications, where risk assessment and intervention strategies could be tailored based on individuals’ personality profiles and associated cognitive mechanisms.

Educational and training programs could particularly benefit from these findings. Decision-making curricula could be adapted to address personality-specific tendencies and cognitive biases, potentially enhancing the effectiveness of financial literacy education and professional development programs. For example, training programs might incorporate different approaches for individuals with high extraversion (focusing on both behavioral tendencies and confidence calibration) versus those with high negative emotionality (emphasizing confidence-building and risk perception accuracy). These interventions could specifically target attention bias patterns, helping individuals recognize when their personality traits may lead to selective information processing during decision-making tasks. These adaptations could lead to more effective learning outcomes and better decision-making capabilities across various contexts.

To implement these insights effectively, practitioners should consider: (1) incorporating appropriate personality assessment tools in their practice, (2) developing targeted intervention strategies based on individual personality profiles, and (3) regularly evaluating the effectiveness of personality-aware approaches. While these applications show promise, future research should validate their effectiveness in specific applied contexts while considering cultural and environmental factors that might influence their implementation.

### 4.4 Limitation and further study

Several limitations of the current study should be acknowledged. Although our study combined self-report measures with behavioral experiments, which helps minimize common method bias, the relatively small sample size (*N* = 110) may limit statistical power for detecting more complex interaction effects or subtle demographic differences (Giner-Sorolla et al., 2024). While our cross-sectional design provided initial evidence for the relationships between personality traits and risky decisions, longitudinal data would be necessary to establish temporal precedence and causal relationships more definitively (Ployhart and Vandenberg, 2010). Additionally, our sample consisted predominantly of university students, which may limit the generalizability of our findings to broader populations with different educational backgrounds and age ranges (Henrich et al., 2010). Although our behavioral experiments provided objective measures of risk-taking, the laboratory setting may not fully capture real-world decision-making processes, where multiple contextual factors could influence risk behavior.

An important limitation concerns our focus on individual-level factors without adequate consideration of situational influences on risk decision-making. Research consistently demonstrates that risk behavior emerges from the interaction between individual characteristics and situational factors (Sartori et al., 2017), yet our laboratory-based design did not systematically vary contextual elements that might moderate personality-risk relationships. Real-world decision-making occurs within complex social, economic, and cultural contexts that may significantly influence how personality traits manifest in risk behavior. The absence of situational manipulations limits our ability to understand when and under what conditions personality-overconfidence-risk pathways are most pronounced.

Future research could address these limitations in several ways. Longitudinal studies with larger and more diverse samples could examine how personality-risk relationships develop over time and how cognitive mechanisms evolve in this process (Bleidorn et al., 2019). Cross-cultural validation studies would be valuable, as cultural factors may moderate the relationships between personality traits and risky decisions (Gebauer et al., 2015). Critically, future research should incorporate systematic investigation of situational moderators, examining how contextual factors such as social pressure, time constraints, and decision stakes influence the personality-overconfidence-risk pathways identified in our study. While our study focused on overconfidence as a mediating mechanism, future research could investigate additional cognitive processes such as regulatory focus (Higgins, 1997) and temporal discounting (Odum, 2011). Additionally, studies could examine the specific attention bias mechanisms suggested by our findings, using eye-tracking or other process-tracing methods to directly assess how personality traits influence information processing during risky decisions. Ecological momentary assessment methods could help capture decision-making processes in natural settings (Hofmann et al., 2014), complementing laboratory-based behavioral measures. Such methodological advances could provide a more comprehensive understanding of how personality traits influence risk-taking behavior across different contexts and populations.

## 5 Conclusion

This study examined the mediating role of overconfidence in the relationship between personality traits and risk decision-making. Through behavioral experiments and psychological measurements, we identified two distinct patterns in personality-risk relationships. Extraversion influences risk-taking through both direct and indirect pathways, with overconfidence serving as a partial mediator. In contrast, Negative Emotionality’s effect on risky decisions operates primarily through overconfidence, demonstrating full mediation. These findings advance our understanding of how personality traits influence decision-making behavior through cognitive mechanisms. The differential mediation patterns suggest that various personality dimensions may shape risky decisions through distinct pathways. This integrated perspective has implications for understanding individual differences in decision-making processes, particularly in contexts where accurate risk assessment is crucial.

## Data Availability

The raw data supporting the conclusions of this article will be made available by the authors, without undue reservation.
