# Supplementary material for: Differential pathways from personality to risk-taking: how extraversion and negative emotionality shape decision-making through overconfidence
Source: Front Psychol. 2025 Jul 28;16:1537658. doi: 10.3389/fpsyg.2025.1537658 (PMC12336267; doi:10.3389/fpsyg.2025.1537658)
Supplement: Supplementary file 1 [file Supplementary_file_1.docx]

## Appendix

### Appendix 1. General Knowledge Questionnaire

The traditional GKQ includes the following ten general knowledge items:

1) Length of the Nile River in kilometers (6,650 km)

2) Einstein's birth year (1879)

3) Depth of the deepest known ocean trench in meters (11,034 m)

4) Year World War I began (1914)

5) Year of first human moon landing (1969)

6) Total length of the Great Wall in kilometers (21,196 km)

7) Years since the pyramids were built (approximately 4,500 years)

8) Year the first motion picture was made (1895)

9) Year DNA's double helix structure was discovered (1953)

10) Operations per second of the first electronic computer (5,000)

Considering cultural differences and contemporary relevance, we developed a culturally adapted version more appropriate for modern Chinese participants:

1) Age at death of Li Hongzhang, late Qing Dynasty statesman (79 years)

2) PetroChina's annual revenue in 2018 (2.35 trillion RMB)

3) World population as of January 2018 (approximately 7.59 billion)

4) McDonald's ranking in Fortune Global 500 brands 2018 (10th)

5) Nike's ranking in Fortune 500, 2018 (341st)

6) Cancer deaths in China, 2018 (approximately 2.56 million)

7) Influenza cases in China, 2018 (760,000)

8) Number of OPEC member countries (13)

9) Mozart's birth year (1756)

10) Napoleon's birth year (1769)

The standardized procedure for calculating overconfidence scores follows these steps:

1) Participants provide an interval estimate (upper and lower bounds) for each item;

2) Instructions explicitly state that participants should be 90% confident that the true value falls within their specified interval;

3) Scoring criteria: 0 points if the true value falls within the provided interval; 1 point if the true value falls outside the provided interval.

4)Sum of points across all ten items yields the total overconfidence score (theoretical range: 0-10)

### Appendix 2. Distribution for risk seeking score and overconfidence score


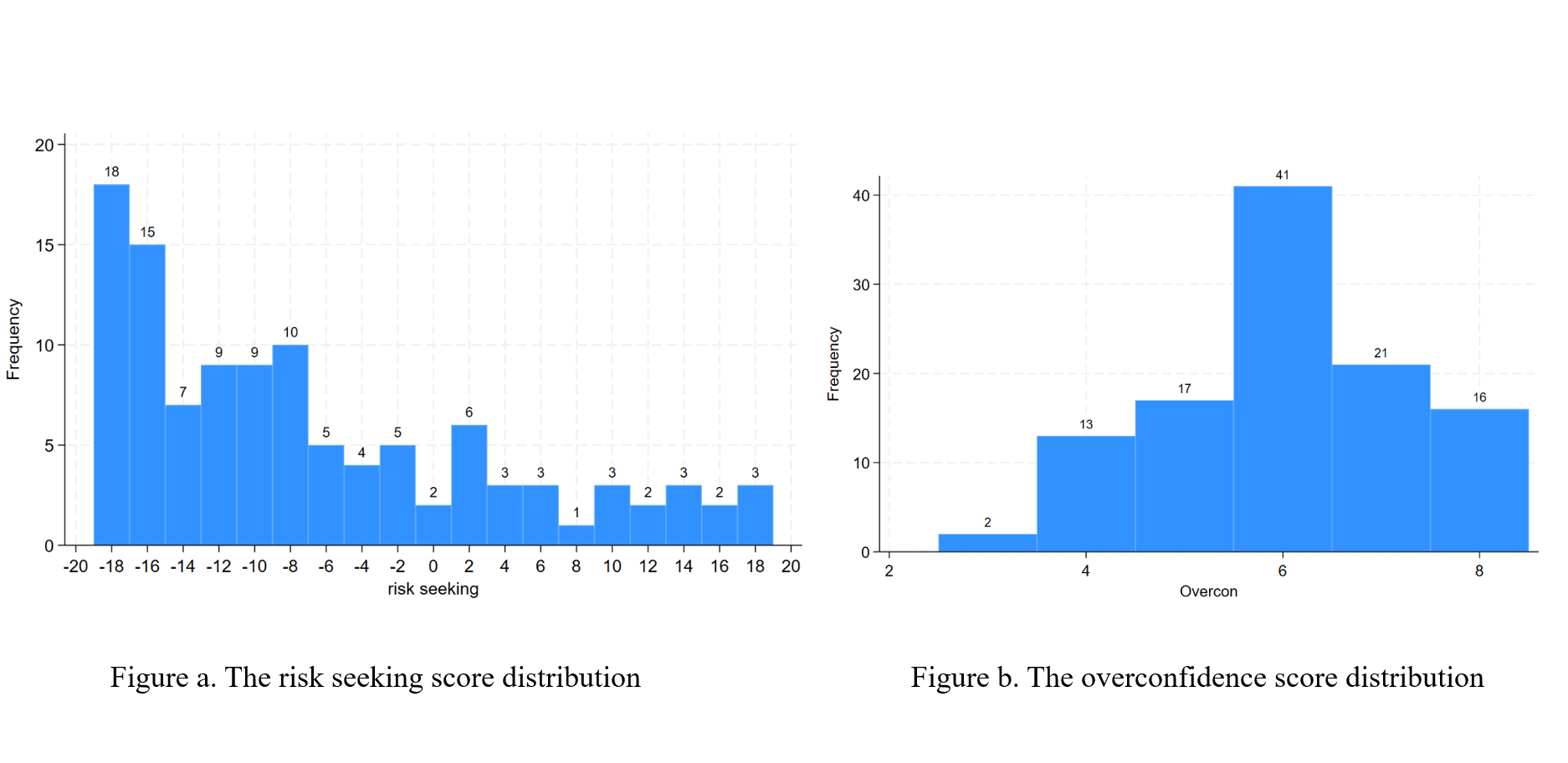


### Appendix 3. Mediation analysis regression result table

| Table A. Simple mediation analysis results | | | | | | | | | | |
| --- | --- | --- | --- | --- | --- | --- | --- | --- | --- | --- |
| Variables | Mediator | Dependent variable | Path | β | t | Effect | SE | BootLLCI | BootULCI | Effect ratio(%) |
| Extraversi-on | Overconfi-dence | Risk seeking | a×b | 0.036* | 1.994 | 0.101* | 0.056 | 0.012 | 0.228 | 20.400 |
|  |  |  | c' | 0.393** | 2.644 | 0.393** | 0.149 | 0.098 | 0.687 | 79.500 |
|  |  |  | c | 0.494*** | 3.631 | 0.499*** | 0.152 | 0.192 | 0.795 | 100.000 |
| Negative Emotiona-lity | Overconfi-dence | Risk seeking | a×b | -0.070*** | -3.538 | -0.216*** | 0.070 | -0.364 | -0.091 | 72.800 |
|  |  |  | c' | -0.081 | -0.476 | -0.081 | 0.170 | -0.417 | 0.256 | 27.100 |
|  |  |  | c | -0.297* | -1.727 | -0.297* | 0.172 | -0.637 | 0.043 | 100.000 |
|  | | | | | | | | | | |
